# Objective sleep efficiency links to cortisol stress recovery via dorsolateral prefrontal-hippocampal regulation

**DOI:** 10.1017/S0033291726104206

**Published:** 2026-05-05

**Authors:** Xiao Luo, Xiaolin Zhao, Yadong Liu, Yina Ma, Yipeng Ren, Zhenni Wei, Zihan Tang, Kaige Guo, Jiahao Luo, Juan Yang

**Affiliations:** 1 Faculty of Psychology, Southwest University, Chongqing 400715, China; 2 Key Laboratory of Cognition and Personality, Ministry of Education, Southwest University, Chongqing 400715, China; 3 West China Institute of Children’s Brain and Cognition, Chongqing University of Education, Chongqing 400715, China; 4 Department of Neurology, University of Groningen, University Medical Center Groningen, 9700 AB Groningen, The Netherlands; 5 State Key Laboratory of Cognitive Neuroscience and Learning, McGovern Institute for Brain Research, Beijing Normal University, Beijing 100875, China; 6 University of Groningen, 9700 AB Groningen, The Netherlands; 7 Chongqing City Vocational College, Chongqing 402160, China

**Keywords:** cortisol stress recovery, dlPFC, hippocampus, objective sleep efficiency, gPPI, FC, fMRI, acute stress, ScanSTRESS, mediation, dlPFC-hippocampus regulation, sleep

## Abstract

**Background:**

Existing evidence highlights sleep’s critical role in regulating cortisol stress recovery; the underlying neural pathways remain unclear. To address this gap, the current study aims to elucidate the neurobiological pathway linking objective sleep efficiency to cortisol stress recovery using functional magnetic resonance imaging (fMRI), with a focus on the functional connectivity (FC) between prefrontal cortex (PFC) and hippocampus.

**Methods:**

Seventy-seven participants completed an acute stress task during a task-dependent and resting-state fMRI scanning. Salivary samples were collected and analyzed as an indicator of cortisol stress recovery. Objective sleep efficiency was measured the night before the fMRI scanning. Using Seed-based gPPI and resting-state FC analysis, we examined the mediating role of PFC-hippocampus FC in the association between objective sleep efficiency and cortisol stress recovery, both during the stress task and in the post-stress resting-state.

**Results:**

Objective sleep efficiency was significantly related to cortisol stress recovery but not with cortisol reactivity. Neurologically, higher sleep efficiency was linked to enhanced prefrontal activity and increased the left dlPFC-hippocampus FC during the acute stress task. Importantly, objective sleep efficiency promoted cortisol stress recovery by the weakened resting-state left dlPFC-hippocampus FC.

**Conclusions:**

This study highlights the pivotal role of left dlPFC-hippocampus regulation underlying sleep’s effect on HPA axis recovery to acute stress. These results suggest a model whereby high objective sleep efficiency promotes adaptive stress recovery through dynamic reallocation of neural resources across acute stress process, characterized by task-dependent coupling and post-stress decoupling of frontal-hippocampal circuitry.

## Introduction

Adequate and effective sleep plays a critical role in maintaining the normal functioning of the neuroendocrine systems, thereby enabling individuals to respond effectively to stressful situations (Girardeau & Lopes-dos-Santos, 2021; Van Dalfsen & Markus, 2018). This benefit is achieved through sleep’s regulatory effects on hypothalamic–pituitary–adrenal (HPA) axis activity (Irwin, 2015; McEwen, 2007; Xie et al., 2013). Consistent with these mechanisms, prior research has demonstrated that sleep disturbances or deprivation can significantly alter the HPA axis activity, often resulting in dysregulation of the cortisol stress response (Jackowska, Fuchs, & Klaperski, 2018; Minkel et al., 2014; Räikkönen et al., 2010; Vargas & Lopez-Duran, 2017).

Importantly, the cortisol stress response exhibited a distinct temporal trajectory, with cortisol levels peaking approximately 20–30 min after the onset of an acute stressor (cortisol reactivity), and subsequently declining to baseline levels (cortisol recovery) (Dickerson & Kemeny, 2004). Reactivity reflects the initial HPA axis response to a challenge, while recovery, the return to baseline following stress, serves as a critical index of regulatory capacity and allostatic load (Degering et al., 2023; McEwen, 2007). Given the high prevalence of chronic cardiovascular disease, burnout, and other stress-related diseases, some researchers argue that failure to recover from stress responses may be a more significant predictor of long-term health than the initial magnitude of reactivity (He, Fan, & Yang, 2021). Consistent with this view, a recent study examining the effects of sleep on cortisol responses to psychological challenges found that poor sleep predicted slower cortisol stress recovery but did not significantly affect cortisol stress reactivity (Zhao et al., 2023).

From a neurological perspective, sleep’s regulatory influence on acute HPA axis activity may stem from its underlying neural mechanisms, including prefrontal cortex (PFC) function and hippocampal interactions (Krause et al., 2017; Motomura, Katsunuma, Yoshimura, & Mishima, 2017; Nowak et al., 2020; Robinson, Erath, Kana, & El-Sheikh, 2018). While existing evidence highlights sleep’s critical role in regulating cortisol stress recovery, no study has yet explored the mediating role of PFC function and its interaction with the hippocampus in the relationship between sleep and cortisol stress recovery. This gap is partly due to the methodological limitations in experimental design. Although task-dependent activation of the PFC and its interaction with the hippocampus during an acute stress task could serve as an indicator of the neural mechanisms underlying acute stress reactivity (Goldfarb et al., 2020; Liu et al., 2023), these measures may not adequately capture the dynamics of acute stress recovery (Broeders et al., 2021; Hermans, Henckens, Joëls, & Fernández, 2017). Therefore, the current study aims to elucidate the neurobiological pathway linking sleep to cortisol stress recovery by employing task-dependent and resting-state functional magnetic resonance imaging (fMRI), with a focus on the functional connectivity (FC) between PFC and hippocampus.

In the current study, an adapted ScanSTRESS paradigm (Streit et al., 2014), validated in prior studies (Liu et al., 2023; Van Oort et al., 2017), was used to induce uncontrollability and social evaluative threat during acute stress. Moreover, this study focused on objective sleep efficiency for two key reasons. First, sleep efficiency, which captures both sleep latency and wakefulness aspects, serves as a precise indicator of sleep quality (Gruber et al., 2014). Second, objective sleep measurement (e.g. actigraphy) exhibits less bias than subjective sleep reports (e.g. sleep log) when estimating sleep variables (Williams et al., 2020).

The prefrontal vulnerability hypothesis emphasizes the heightened susceptibility of the PFC to sleep loss (Horne, 1993; Krause et al., 2017), particularly in the dorsolateral PFC (dlPFC) and ventromedial PFC (vmPFC). These regions are critical for adaptive stress coping and cortisol response regulation (Liu et al., 2023; Maier, Makwana, & Hare, 2015; Sinha, Lacadie, Constable, & Seo, 2016). Besides that, a key neural mechanism through which the PFC modulates HPA axis recovery involves its interaction with the hippocampus, a well-established regulator of the HPA axis, rich in glucocorticoid receptors, and vital for inhibiting HPA axis activity during the post-stress recovery via negative feedback (Jacobson & Sapolsky, 1991; Sapolsky, Krey, & McEwen, 1984; Surget et al., 2011). Particularly, the dlPFC has been proven to modulate hippocampal function through direct and indirect pathways(Li et al., 2022; Schneider et al., 2017).

More importantly, the reciprocal resource allocation mode further posits a competitive relationship between dlPFC and hippocampus during the dynamic reallocation of neural resources across the acute stress process (Hermans, Henckens, Joëls, & Fernández, 2014; Hermans et al., 2011). Based on this framework, we propose that objective sleep efficiency may exert opposing effects on the efficacy of the dlPFC-hippocampus circuit, depending on whether this circuit is engaged in a stress task or in a post-stress rest. Specifically, we hypothesized that the dlPFC-hippocampus circuit during the resting-state following stress task may serve as a mediator of the association between objective sleep efficiency and cortisol stress recovery.

## Materials and methods

### Participants

Seventy-seven participants were recruited by advertisement in a local university (37 females; mean age = 20.18 ± 1.97 years, range = 18–26). All participants’ eligibility, current health status, and health behaviors were ascertained using their self-reports. Exclusion criteria were acute or chronic psychiatric or somatic diseases, psychotropic or glucocorticoid medication intake, and alcohol/drug abuse. Female participants were tested during their luteal phase and did not use oral contraceptives leading up to the experiment (Kajantie & Phillips, 2006). Finally, 74 and 71 participants were included in the analysis of task- and resting-state fMRI, respectively, to maximize the use of data.

All participants provided written informed consent and were informed that they could withdraw at any time. All protocols were approved by the local institutional review board and complied with the standards of the Declaration of Helsinki.

### General experimental procedure

**Figure 1.**
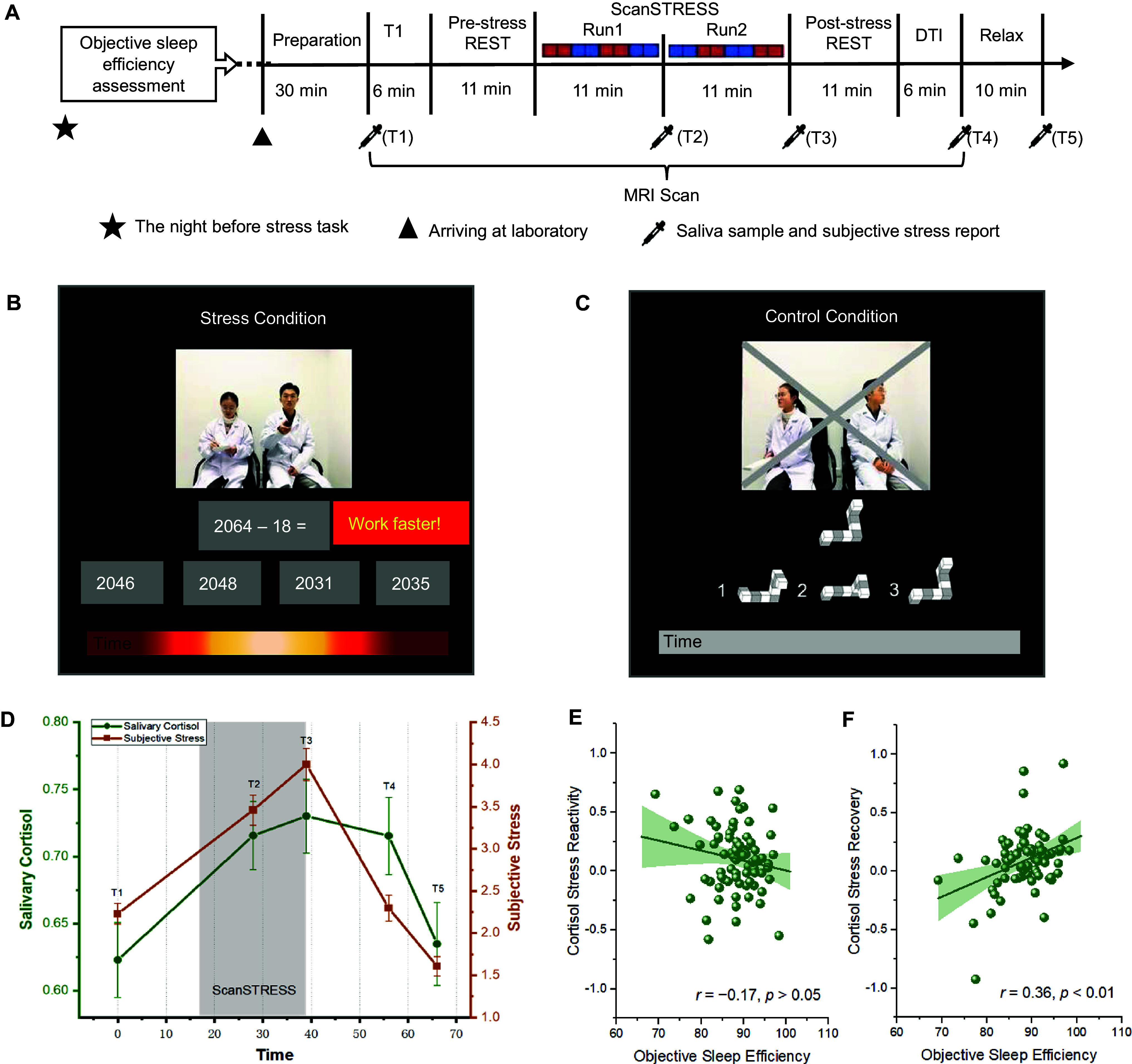
Experimental procedure and behavioral results. (a) Participants were first asked to record their sleep by actigraphy and sleep log the night before the formal acute stress experiment. After arriving at the laboratory the next day, participants were asked to rest for 30 min before entering the MRI scanner. During the scanning, a T1 image was acquired first, followed by a resting-state image. Immediately, the ScanSTRESS paradigm was used to induce a stress response for 22 min. The order for conditions is counter-balanced in two runs, with the red representing the stress block and the blue representing the control block. Then, there was another resting-state scan and structural scan; lastly, participants were debriefed for 10 min before they left the laboratory. During this period, participants provided five saliva samples. (b) In the stress condition of the ScanSTRESS, participants were asked to solve challenging cognitive tasks (serial subtraction and mental rotation) under time pressure (red bar) and social evaluative threat. (c) In the control condition, participants went through similar, although much easier tasks (find the matched figure or number) without time constraints (gray bar), negative feedback, and social evaluative threat. (d) Salivary cortisol secretion and subjective stress report during the experiment. Time significantly affected subjective stress self-reports (*F* (4, 73) = 84.40, *p* < 0.001, *η*^2^_p_ = 0.54) as well as salivary cortisol levels (*F* (4, 73) = 6.95, *p* < 0.001, *η*^2^_p_ = 0.08), the ScanSTRESS paradigm successfully induced participants’ subjective and cortisol stress responses. (e) The correlation between objective sleep efficiency and cortisol stress reactivity was nonsignificant (*r* = −0.17, *p* > 0.05), while (f) objective sleep efficiency was positively related to participants’ cortisol stress recovery (*r* = 0.36, *p* = 0.001). The solid line represents the least-squares fit, and the shading represents the 95% CI of the linear fit.

We first informed the participants how to use actigraphy and finish the sleep log, and measured participants’ objective sleep efficiency by actigraphy and sleep log the day before the formal acute stress experiment (Figure 1a). After arriving at the laboratory on the second afternoon, participants were asked to rest for 30 min before entering the MRI scanner. During the scanning, a T1 image (6 min) was acquired first, followed by a resting-state image (11 min). Immediately, the ScanSTRESS paradigm was used to induce a stress response for 22 min. In the stress condition (Figure 1b), participants performed tasks that challenged serial subtraction and mental rotation abilities under time pressure. Two investigators in laboratory coats gave disapproving feedback when the participants answered wrongly or slowly on the live video stream. In the control condition (Figure 1c), participants performed figure- and number-matching tasks without time pressure or feedback. The order for conditions is counter-balanced in two runs, with the red representing the stress block and the blue representing the control block. Then, there was another resting-state scan (11 min) and a DTI scan (6 min); lastly, participants were debriefed for 10 min before they left the laboratory. Saliva samples and subjective stress reports were collected from the participants at five critical time points: before entering the scanner, immediately after ScanSTRESS run 1, after ScanSTRESS run 2, after scanning, and before leaving. To control for circadian rhythms in cortisol, all acute stress tasks were performed between 1:00 pm and 5:30 pm.

### Objective sleep efficiency assessment

Objective sleep parameters were assessed using wrist-worn actigraphy (wGT3X-BT, Pensacola, USA), with data collected at a 60-second sampling epoch. The analysis interval for actigraphic sleep was defined using sleep log data, specifically as the time between trying to sleep and finally getting up. Within this log-defined interval, sleep and wake states for each epoch were scored automatically using the Cole-Kripke algorithm in ActiLife software (v6.13.4) (Cole et al., 1992). Objective sleep duration was derived as the time between trying to sleep and getting up from sleep log data, minus the time of sleep latency and wakefulness after sleep onset from the ActiLife software. Objective sleep efficiency was then calculated as (objective sleep duration/the time between trying to sleep and getting up) × 100%.

### Salivary cortisol data acquisition and analyses

Saliva samples were collected using a sampling device (Salivette, Sarstedt, Germany), and all saliva samples were stored at −20 °C until analysis. Cortisol concentrations were analyzed using an enzyme-linked immunosorbent assay kit (IBL-Hamburg, Germany). The sensitivity of the cortisol assay was 0.005 μg/dL, and the inter-assay coefficient of variation for the cortisol assay was 11.56%. Cortisol values were log10-transformed to ensure a normal distribution and extreme values beyond ±3 standard deviations were replaced by critical values. Following previous suggestions (He et al., 2021; Zhao et al., 2023), we further calculated the difference between peak (T3) and baseline salivary cortisol values (T1) as an indicator of cortisol stress reactivity, and the difference between peak (T3) and last measured values (T5) as an indicator of cortisol stress recovery.

### Image data acquisition and preprocessing

Functional and anatomical whole-brain images were acquired on a 3 T Siemens Trio MRI scanner (Munich, Germany). We acquired 331 volume-functional images from each participant during the ScanSTRESS task, using a T2-weighted gradient echo-planar imaging sequence [repetition time (TR), 2000 ms; echo time (TE), 30 ms; slice thickness, 2 mm; matrix, 64 × 64; field of view (FOV), 224 × 224 mm^2^; voxel size, 2 × 2 × 2 mm^3^]. High-resolution T1-weighted three-dimensional (3D) fast-field echo sequences were obtained for anatomical reference (slices, 192; TR, 2530 ms; TE, 2.98 ms; slice thickness, 1 mm; FOV, 256 × 256 mm^2^; voxel size, 0.5 × 0.5 × 1 mm^3^; flip angle = 7°). We also acquired 235 resting-state functional images (TR, 2000 ms; TE, 30 ms; slice thickness, 2 mm; FOV, 224 × 224 mm^2^; voxel size, 2 × 2 × 2 mm^3^).

Preprocessing of the task fMRI data was done using DPABI (a toolbox for Data Processing & Analysis for Brain Imaging, http://rfmri.org/dpabi) (Yan, Wang, Zuo, & Zang, 2016). First, the three-dimensional DICOM image data of each subject were converted to the four-dimensional NIFTI format. The first 10 volumes were discarded to stabilize the magnetic resonance signal. The remaining images were corrected for slice acquisition timing, realigned for six head motion parameters correction, spatially normalized into the standard Montreal Neurological Institute (MNI) space in 2 × 2 × 2 mm^3^ voxel sizes, and smoothed by convolving a 4-mm isotropic three-dimensional Gaussian kernel. Three participants were excluded due to excessive head movement during task-dependent fMRI scan (mean framewise displacement (FD) > 0.2)(Jenkinson, Bannister, Brady, & Smith, 2002).

### Task-evoked whole-brain activation analysis and gPPI analysis

The preprocessed data were further analyzed using SPM12. T-contrast (stress vs control) was used to obtain the neural brain activity induced by acute psychological stress. Subsequently, whole-brain regression analyses were performed with objective sleep efficiency as the independent variable while controlling for age and sex. Significant clusters were determined at a height threshold of *P* < 0.001 at the voxel level and an extent threshold of *P*_FDR_ < 0.05 at the corrected whole-brain cluster level.

Task-based generalized physiological–psychological interaction (gPPI) functional connectivity analysis was performed using the CONN toolkit (https://www.nitrc.org/projects/conn/). We used the significant PFC clusters identified in the whole-brain regression analyses above as seeds, focusing on their functional connectivity with the bilateral hippocampus (defined by the AAL template). Subsequently, regression analyses were performed using objective sleep efficiency as an independent variable while controlling age, sex, and FD, corrected for multiple comparisons using an *p*_FDR_ < 0.05 threshold. Significant functional connectivity strength values were then extracted for subsequent mediation analyses.

### Resting-state FC analysis

Pre-stress and post-stress resting-state data were analyzed using seed-based functional connectivity analysis with dlPFC and bilateral hippocampus seeds with the CONN toolbox version 19c (Whitfield-Gabrieli & Nieto-Castanon, 2012). Preprocessing for resting-state images was performed using the CONN FC toolbox, which included slice-timing correction, realignment and unwarp and susceptibility distortion correction using the creation of a voxel-displacement map, co-registration, outlier detection (ART-based identification of outlier scan for scrubbing), segmentation, normalization to the EPI template with a resampling voxel size of 2.0 × 2.0 × 2.0 mm^3^, and smoothing with an 8 mm full-width half-maximum Gaussian kernel. The CONN toolbox-featured intermediate scrubbing parameters were used to compute head motion in each session of each participant. In the next step, we evaluated the sessions in which head motion exceeded a threshold in more than 25% of volumes. This head motion criterion resulted in the exclusion of six participants’ rest sessions. The pre-processed EPI data were then used and denoised within each scan, run through demeaning, linear detrending, and bandpass filtering at 0.008–0.09 Hz. The regression of the first principal component from a CompCor was performed to remove white matter and CSF confounds, along with six motion parameters.

In the first-level analysis, the individual-level post-stress resting-state FC maps were entered into the design matrix, focused on the FC between the left dlPFC (saved from whole-brain activation cluster) and the hippocampus (defined by AAL template). Second-level multiple regression analysis was done with objective sleep efficiency as the covariate of interest while including sex, age, and FD as nuisance variables, corrected for multiple comparisons using an *p*_FDR_ < 0.05 threshold. Parameter estimates were extracted from significant clusters. Importantly, we also examined the association between objective sleep efficiency and PFC-hippocampus FC during a pre-stress resting-state scan to determine whether this relationship reflects a general correction or is specific to the post-stress period.

### Mediation analysis

The mediation models and statistical tests were further examined using PROCESS 2.1.6 in SPSS version 25 (IBM Corp., Armonk, NY). The significance of the indirect or mediated effect was assessed using 5000 bias-corrected bootstraps. The indirect effect (c − c’) was considered significant if the 95% confidence interval (CI) did not include zero.

## Results

### Objective sleep efficiency links to cortisol stress recovery but not reactivity

The ScanSTRESS paradigm successfully induced participants’ subjective and cortisol responses to acute stress (Figure 1d). Regarding the subjective reports of stress levels, the effect of Time was significant (*F* (4, 73) = 84.40, *p* < 0.001, *η*^2^_p_ = 0.54), as participants’ subjective stress levels increased significantly after the beginning of stress exposure (*p*_time2 − time1_ < 0.001, 95% CI: [0.944, 1.516]), attained the highest levels immediately after stress induction (*p*_time3 − time2_ < 0.001, 95% CI: [0.294, 0.787]), and decreased significantly at multiple points in the recovery phase (*p*_time3 − time4_ < 0.001, 95% CI: [1.385, 2.021]; *p*_time4 − time5_ < 0.001, 95% CI: [0.487, 0.892]). The effect of time was also significant for the salivary cortisol levels (*F* (4, 73) = 6.95, *p* < 0.001, *η*^2^_p_ = 0.08). Post-hoc analysis revealed that participants’ salivary cortisol levels increased significantly once stress began (*p*_time2 − time1_ < 0.01, 95% CI: [0.039, 0.146]; *p*_time3 − time1_ < 0.01, 95% CI: [0.046, 0.168]), remained a high level after stress exposure, and then decreased significantly in the recovery phase (*p*_time3 − time5_ < 0.01, 95% CI: [0.042, 0.149]; *p*_time4 − time5_ < 0.01, 95% CI: [0.036, 0.125]). Importantly, objective sleep efficiency was not significantly related to cortisol stress reactivity (*r* = −0.17, *p* > 0.05, Figure 1e), but was positively related to cortisol stress recovery (*r* = 0.36, *p* = 0.001, Figure 1f). Steiger’s *Z*-test further confirmed that the association between sleep efficiency and cortisol stress recovery is significantly stronger than reactivity (*z* = 3.72, *p* < 0.001).

### Objective sleep efficiency links to task-evoked prefrontal activity and FC between dlPFC and hippocampus

**Figure 2.**
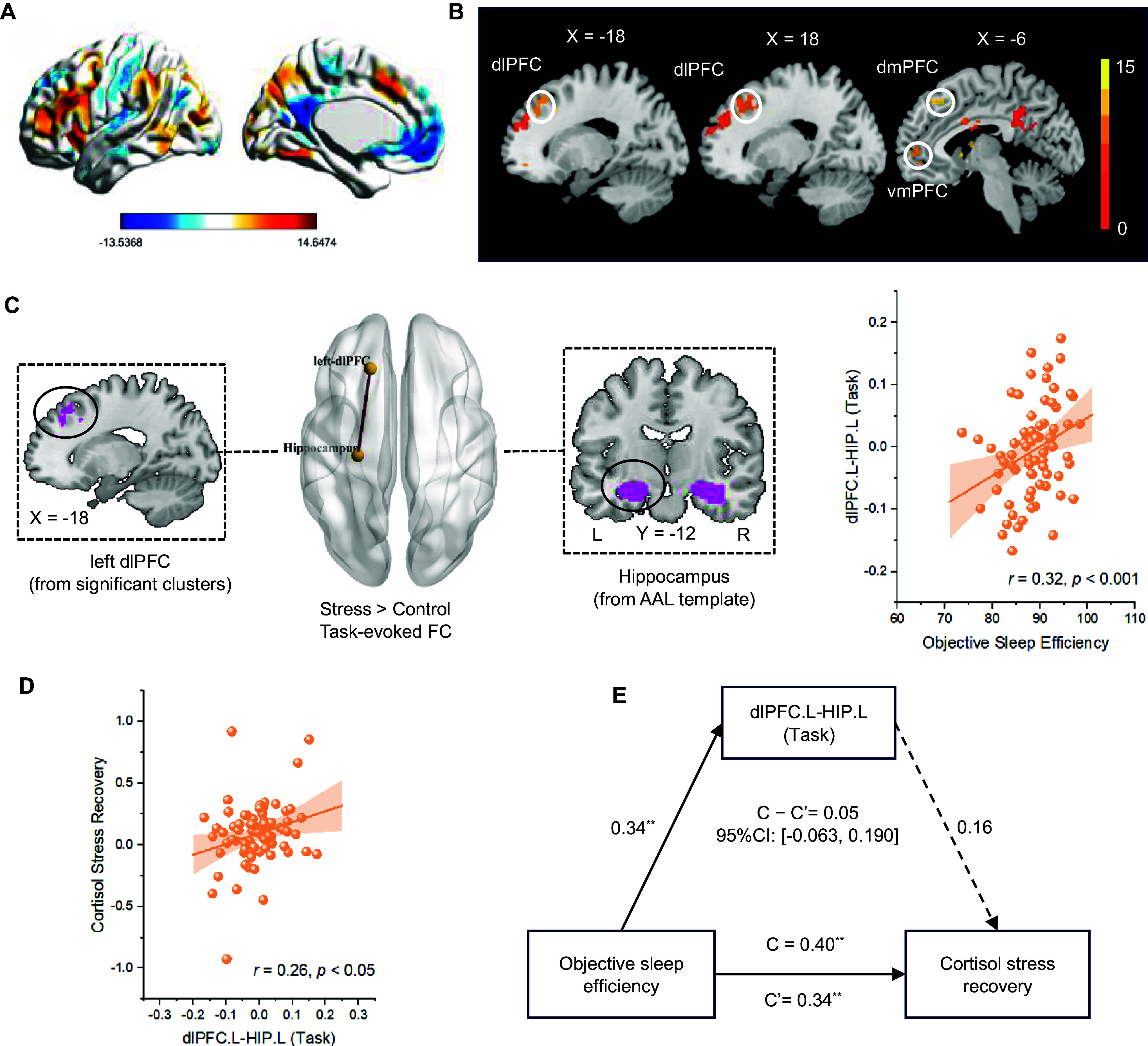
Task fMRI results. (a) Neural response to the ScanSTRESS paradigm (stress condition vs control condition; *p*_FDR_ < 0.05 at the cluster level). (b) Objective sleep efficiency was significantly correlated with prefrontal activity during acute stress (stress > control) (corrected: *p*_voxel_ < 0.001, *p*_cluster-FDR_ < 0.05). (c) The generalized Physical-Psychological Interaction (gPPI) analysis showed that objective sleep efficiency was positively associated with the task-dependent functional connectivity (FC) between left dlPFC and left hippocampus (defined by the AAL template) (corrected: *p*_FDR_ < 0.05). (d) The task-evoked FC strength of left dlPFC-left hippocampus positively correlated with participants’ cortisol stress recovery (*r* = 0.26, *p* < 0.05). (e) The mediating effect of task-evoked dlPFC-hippocampus FC between objective sleep efficiency and cortisol stress recovery was not significant (indirect effect estimate = 0.05, *SE* = 0.06, 95%CI: [−0.063, 0.190]). * *p* < 0.05, ^**^ *p* < 0.01, ^***^ *p* < 0.001.

We conducted T-contrast (stress vs control) to obtain the neural brain activity induced by acute psychological stress (Figure 2a), and found that objective sleep efficiency was positively correlated with the activity of the PFC, including the bilateral dlPFC, left dmPFC, left vmPFC (Figure 2b).

The gPPI showed that objective sleep efficiency was positively (*r* = 0.32, *p* < 0.001) associated with the task-evoked FC (stress vs control) between left dlPFC and left hippocampus (*t* = 2.84, *p*_FDR_ < 0.05) (Figure 2c). Moreover, this task-evoked FC between left dlPFC and left hippocampus was positively associated with cortisol stress recovery (*r* = 0.26, *p* < 0.05) (Figure 2d). However, it failed to mediate the relationship between objective sleep efficiency and cortisol stress recovery (indirect effect estimate = 0.05, *SE* = 0.06, 95%CI: [−0.063, 0.190]) (Figure 2e).

### Post-stress resting-state FC of dlPFC-hippocampus mediated the association between objective sleep efficiency and cortisol stress recovery

**Figure 3.**
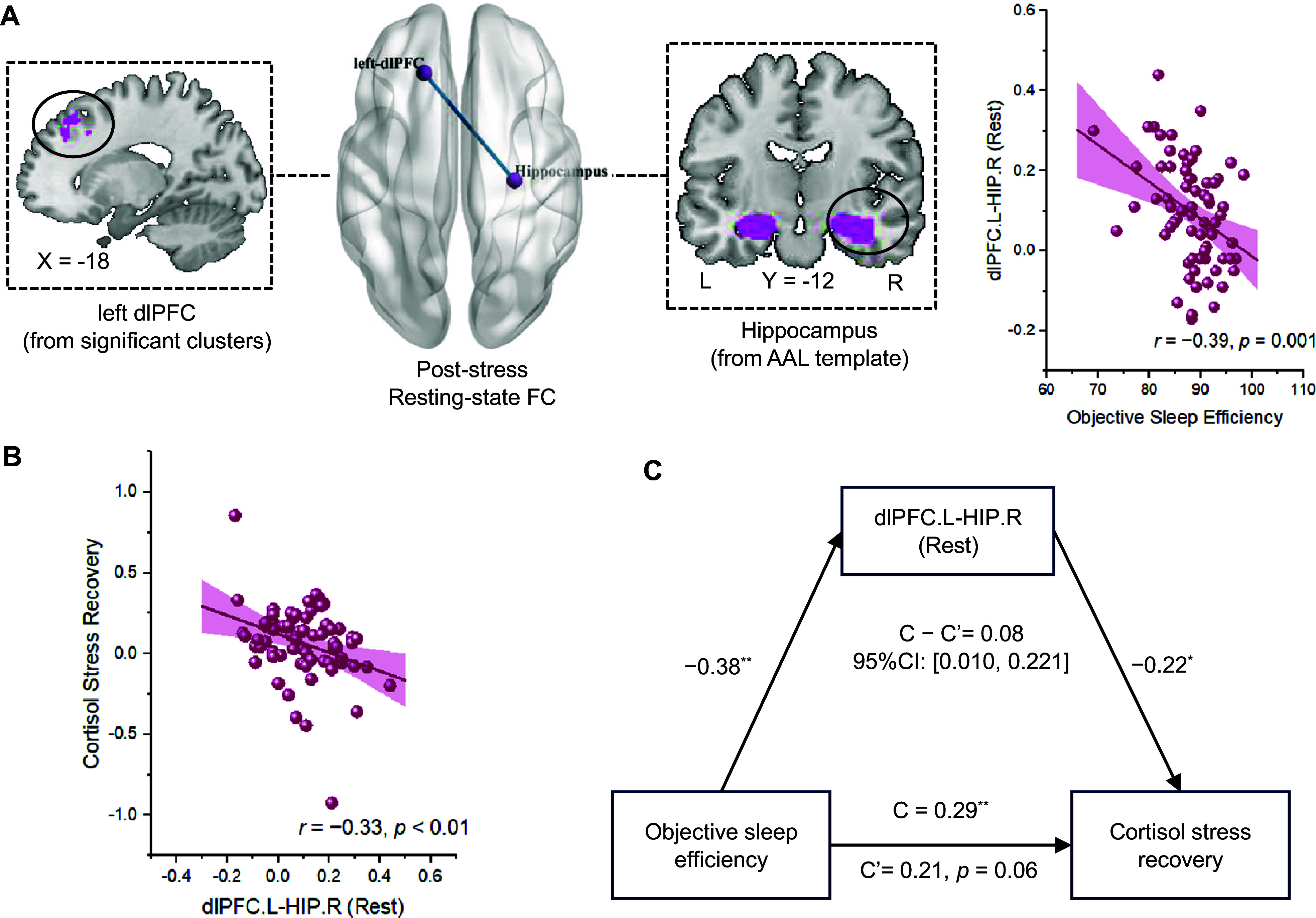
Resting state fMRI results after ScanSTRESS task. (a) Objective sleep efficiency was negatively correlated with the resting-state FC between left dlPFC and right hippocampus after acute stress (corrected: *p*_FDR_ < 0.05). (b) The resting-state FC strength of left dlPFC-right hippocampus after acute stress was negatively related to cortisol stress recovery (*r* = −0.33, *p* < 0.01), and (c) further mediated the effect of objective sleep efficiency on cortisol stress recovery (indirect effect estimate = 0.09, *SE* = 0.05, 95%CI: [0.010, 0.221]). * *p* < 0.05, ^**^ *p* < 0.01, ^***^ *p* < 0.001.

Regression analyses with age and sex controlled revealed that objective sleep efficiency was negatively (*r* = −0.39, *p* = 0.001) related to post-stress resting-state FC of left dlPFC-right hippocampus (*t* = −3.42, *p*_FDR_ < 0.01) (Figure 3a). We found that this resting-state left dlPFC-right hippocampus FC was also negatively associated with participants’ cortisol stress recovery (*r* = −0.33, *p* < 0.01) (Figure 3b). Furthermore, the results verified a mediating pathway with higher objective sleep efficiency initially linked to lower resting-state connectivity of left dlPFC-right hippocampus, which then related to higher cortisol stress recovery (indirect effect estimate = 0.09, *SE* = 0.05, 95%CI: [0.010, 0.221]) (Figure 3c).

Importantly, pre-stress regression analyses showed that objective sleep efficiency was not significantly (*r* = −0.12, *p* > 0.05) related to pre-stress resting-state FC of left dlPFC-right hippocampus (*t* = −0.98, *p*_uncorrected_ > 0.05), manifesting that the association between objective sleep efficiency and PFC-hippocampus FC is specific to the post-stress period.

## Discussion

The present study integrated actigraphy, fMRI, and salivary cortisol measures to investigate the neuroendocrine pathways linking objective sleep efficiency to acute stress recovery. Our findings revealed that objective sleep efficiency was positively associated with cortisol stress recovery but not with cortisol reactivity. Neuroimaging data further demonstrated that objective sleep efficiency exerted opposing effects on the functional efficacy of the dlPFC-hippocampus circuit. Specifically, higher objective sleep efficiency was linked to greater dlPFC activation and increased FC between the left dlPFC and hippocampus during stress exposure. Conversely, higher objective sleep efficiency predicted reduced FC within the same circuit (left dlPFC-right hippocampus) during the post-stress resting state, which in turn was associated with greater cortisol recovery. Together, these results suggest a model whereby high objective sleep efficiency promotes adaptive stress recovery through dynamic reallocation of neural resources across the acute stress process, characterized by task-dependent coupling and post-stress decoupling of frontal-hippocampal circuitry.

While there has been a steady increase in research exploring the relationship between sleep and stress, findings regarding the impact of sleep on acute stress response have remained inconsistent. Studies have reported divergent patterns in salivary cortisol stress responses, including elevated, blunted, or unchanged levels (Jackowska et al., 2018; Minkel et al., 2014; Räikkönen et al., 2010; Van Dalfsen & Markus, 2018). Researchers have proposed that these mixed findings may stem from confounding factors, such as cortisol stress response (Zhao et al., 2021). Specifically, cortisol reactivity and recovery represent two distinct dimensions of the stress response process, each with unique utility in describing individuals’ allostatic states (McEwen, 2007; Terry, Meng, Huo, & Bartley, 2024). Consistent with our previous work (Zhao et al., 2023), the current study found high objective sleep efficiency links to cortisol stress recovery but not reactivity. Similarly, individuals with poorer sleep have significantly less or slower cardiovascular recovery from stress compared to those with better sleep, although they exhibited similar or even higher reactivity levels (Massar, Liu, Mohammad, & Chee, 2017; Mezick et al., 2014). These findings suggested that higher objective sleep efficiency is related to more effective cortisol stress recovery, a factor that is linked to long-term physical and mental health outcomes (Degering et al., 2023; McEwen, 2007).

With neuroimaging data, we found that objective sleep efficiency related to increased activity in several regions of PFC during ScanSTRESS, a higher-order cognitive task with social evaluative stress (Van Oort et al., 2017), which supported the Prefrontal Vulnerability Hypothesis (Horne, 1993). Among them, dlPFC, vmDFC, and dmPFC are involved in top-down guidance of attention and thought, emotion regulation, and error monitoring, respectively (Arnsten, 2009; Krause et al., 2017), supporting that high sleep efficiency can help individuals maintain more resources in the PFC when facing stress, thus facilitating immediate problem-solving(Van Oort et al., 2017). This was also supported by the results that objective sleep efficiency related to increased FC between left dlPFC and hippocampus during stress task. Existing studies manifested that the coupling between the dlPFC and hippocampus supports working memory (Bilek et al., 2013; Schneider et al., 2017), with voluntary forgetting relying on enhanced inhibition of the hippocampus by the dlPFC (Oehrn et al., 2018). These findings suggested that high sleep efficiency may contribute to acute stress processing by enhanced PFC activity and left dlPFC-hippocampus FC during stress exposure.

Remarkably, lower objective sleep efficiency links to heightened resting-state left dlPFC-hippocampus FC in the recovery phase, which in turn relates to cortisol stress recovery. This indicated that sleep-induced alterations in frontal-limbic systems may indirectly influence the sensitivity of the HPA axis (Van Dalfsen & Markus, 2018). Since the functional coupling between the dlPFC and hippocampus is related to working memory and voluntary forgetting (Bilek et al., 2013; Schneider et al., 2017), although it promoted stress processing in the reactivity phase, it may impede recovery if the participants ruminate or immerse in the task-related reminiscence in the stress recovery phase. Our results show that sleep affects cortisol stress recovery through the FC of left dlPFC-hippocampus, revealing the endocrine regulatory function of this circuit. Previous research provided similar evidence that insufficient sleep may impair the regulatory function of the PFC in the limbic area (Motomura et al., 2017; Nowak et al., 2020), whereas both dlPFC and hippocampus activity associate with decreased HPA axis’s response to acute stress (Jacobson & Sapolsky, 1991; Pulopulos et al., 2020). Importantly, objective sleep efficiency is not generally correlated with resting-state FC in this circuit but is specific to the post-stress recovery period.

Taken together, these results suggest a model whereby higher objective sleep efficiency promotes adaptive stress recovery through dynamic reallocation of neural resources across the acute stress process. Specifically, high objective sleep efficiency supports task-engaged coupling between the dlPFC and hippocampus during stress exposure, enhancing cognitive and regulatory resources for acute challenge resolution (Goldfarb et al., 2020; Schneider et al., 2017). Conversely, the same neural circuit undergoes adaptive decoupling during post-stress recovery, which appears critical for disengaging from stress-related processing and promoting cortisol recovery (Jacobson & Sapolsky, 1991; Pulopulos et al., 2020). This dynamic reallocation of neural resources underscores the role of sleep in calibrating the brain’s flexibility, enabling it to effectively meet demands and then efficiently restore homeostasis.

Our findings underscore the importance of good sleep quality, particularly high sleep efficiency, for neuroendocrine resilience. From an intervention perspective, sleep efficiency is a modifiable target. Cognitive-behavioral therapy for insomnia, sleep hygiene education, and mindfulness-based interventions have proven effective in improving sleep continuity and efficiency (Enomoto et al., 2022; Rusch et al., 2019). Our results suggest that such interventions, by enhancing sleep efficiency, may strengthen the prefrontal-limbic circuits governing stress recovery, thereby potentially reducing the long-term health risks associated with impaired HPA axis regulation. Future research should test this directly by combining sleep interventions with neuroendocrine and neuroimaging assessments to establish causality and explore therapeutic mechanisms.

Some limitations of this study need further investigation in future research. First, objective sleep efficiency was measured only for the night immediately preceding the stress task. While this design captures the proximal effect of sleep on next-day stress physiology, it may reflect night-to-night variability (Zhao et al., 2023). Future studies should employ multi-night actigraphy assessments to derive more stable estimates of habitual sleep efficiency and examine its association with neural and endocrine stress responses. Second, we only collected salivary samples at five key time points. Future studies may benefit from more acquisition of salivary cortisol, especially in the stress recovery phase. Third, the present study relies on samples of healthy college students; whether it can be extended to other groups remains to be further verified. Besides, we utilized a correlational design and were unable to provide causal conclusions regarding the relationship between sleep and cortisol stress recovery. Future studies may consider combining fMRI and tDCS to validate our findings or using a long-term follow-up study design to gain a deeper understanding of their relationship.

## Conclusion

In summary, the present study shows objective sleep efficiency links to cortisol stress recovery rather than stress reactivity, and sheds light on the neurobiological pathways underlying the effect of sleep on cortisol stress recovery from acute stress situations, unveiling the pivotal role of left dlPFC-hippocampus regulation. These findings provide a robust foundation for better interpreting and alleviating stress reactions induced by poor sleep, which may assist in preventing and reducing various health issues stemming from stress.
